# Effects of telerehabilitation-based respiratory and corrective exercises among the elderly with thoracic hyper-kyphosis: a clinical trial

**DOI:** 10.1186/s12877-024-04779-8

**Published:** 2024-03-06

**Authors:** Elham Eftekhari, Rahman Sheikhhoseini, Zahra Salahzadeh, Mahdis Dadfar

**Affiliations:** 1 Department of Corrective Exercise & Sport Injury, Faculty of Physical Education and Sport Sciences, Allameh Tabataba’i University, Tehran, Iran; 2 Department of Corrective Exercise & Sport Injury, Faculty of Physical Education and Sport Sciences, Allameh Tabataba’i University, Western Azadi Sport Complex Boulevard, Hakim Highway, Tehran, Iran; 3 Department of Physiotherapy, Faculty of Rehabilitation Sciences, Tabriz University of Medical Sciences, Tabriz, Iran; 4 Department of Human Health and Performance, Faculty of Kinesiology, University of Houston, Houston, TX USA

**Keywords:** Therapeutic exercises, Posture, Kyphosis, Elderly

## Abstract

**Background:**

Aging is associated with changes in the musculoskeletal system, including increased susceptibility to spine malalignments. Utilizing corrective exercises with a therapeutic emphasis can be beneficial in the elderly with thoracic spine hyperkyphosis.

**Objective:**

This study aimed to investigate the effects of six weeks of telerehabilitation-based respiratory and corrective exercises on quality of life, disability, thoracic kyphosis, craniovertebral angle, shoulder angle, cranial angle, and chest expansion in the elderly with thoracic spine hyperkyphosis.

**Methods:**

In this clinical trial, a total of 40 participants aged 60 and above with thoracic hyperkyphosis were randomly divided into the control (*N* = 20) and experimental (*N* = 20) groups. The experimental group performed the corrective exercises for six weeks (3 sessions per week). The control group performed general stretching exercises during the same time period. We measured the outcomes of quality of life, disability, thoracic kyphosis, craniovertebral angle, shoulder angle, cranial angle, and lung expansion before and after the intervention. Analysis of covariance (ANCOVA) was employed to analyze the data. A *P-value* ≤ *0.05* was considered statistically significant.

**Results:**

Quality of life (*P* < *0.001*, Effect Size (ES): 0.44), chest expansion (*P* < *0.001*, ES: 0.56), thoracic kyphosis angle (*P* < *0.001*, ES: 0.31), craniovertebral (*P* < *0.001*, ES: 0.33), cranial (*P* < *0.001*, ES: 0.38), and shoulder (*P* = *0.005*, ES: 0.20) angles were significantly improved in the experimental group as compared with controls. However, no statistically significant difference was observed between the two groups in terms of physical ability (*P* = *0.251*, ES: 0.04).

**Conclusion:**

It is therefore recommended that online corrective exercises be used in the rehabilitation protocol to improve the quality of life, posture, chest expansion, and disability in the elderly with thoracic kyphosis.

**Supplementary Information:**

The online version contains supplementary material available at 10.1186/s12877-024-04779-8.

## Introduction

According to the World Health Organization’s (WHO) reports, the aging population is projected to increase continuously, reaching 1.5 billion by 2050 [1]. This future trend has various implications on different aspects, especially in societies’ economic and social layers, due to the greater demand for health care services [2]. Among the factors guaranteeing health, musculoskeletal health has significantly affected the elderly population. It has turned into a global-scale priority, and scientists have devised different strategies designed to improve the quality of life and reduce both the pain and disability caused by physiological changes in the aging population [3]. Among musculoskeletal changes, the altered spine alignment, including the tendency to increase thoracic kyphosis (TK), sagittal vertical axis, and pelvic angle, occurs with aging [4]. It is shown that the increased TK may be associated with osteoporosis, weak trunk muscles, and decreased physical activity in the elderly population [5]. It has been suggested that elderly people with Thoracic Hyperkyphosis (THK) have numerous impairments in the musculoskeletal, neuromuscular, and sensory systems as compared with the elderly without THK [6].

THK has consequences beyond the thoracic spine and cage, namely an increase in the pain and risk of shoulder dysfunction, compromised function, and alignment in the cervical, thoracic, and lumbar spine [7]. In addition to forward head posture, increased scapula rotation, decreased lumbar lordosis, and weakness in spinal extensor muscles [7], biomechanical changes like the greater anterior movement of the center of gravity and postural sway impair balance maintenance ability and increase the risk of falling [6, 8]. Additionally, increased TK may lead to respiratory complications like poor respiratory function (such as dyspnea and decreased vital capacity) [5], which is believed to affect the quality of life physiologically and may also increase the mortality rate [8]. Therefore, managing THK may be a plausible approach for preventing more health-related and life-threatening consequences of THK in the aging population. Thus far, several corrective exercise protocols have been designed to avoid the progression of the THK and improve its effects on well-being, posture, and respiratory capacity, including exercises on spinal mobility, occipital and pectoral muscles stretching, and cervical flexor and retractor muscle strengthening [9].

Previous studies recommended that the use of corrective exercises with a therapeutic emphasis can be beneficial in ameliorating THK and managing respiratory dysfunction [10–12]. These protocols include a variety of exercises, such as diaphragmatic breathing, thoracic mobility, stability, strengthening, and stretching [11, 12]. Combining breathing exercises with thoracic muscle exercise routines is also recommended to improve respiratory function in the elderly with mild osteoporosis-related kyphosis [13]. The limitations, such as pain and discomfort, or even world-scale pandemics, such as the COVID-19 pandemic and lockdowns, may prevent older adults from participating in exercise protocols [14]. A trustworthy method of delivering rehabilitation services remotely is telerehabilitation [15]. Today's advancements in communication and information technologies have greatly simplified remote healthcare professionals and patients' connection [16]. Numerous rehabilitation services, including education, monitoring, supervision, prevention, assessment, intervention, and counseling, can be offered by using telerehabilitation [17]. However, when compared to rehabilitation that is provided face-to-face, the effectiveness of telerehabilitation is still unknown [15].

In recent years, it has been demonstrated that using telerehabilitation can result in considerable clinical gains; as a result, it appears to be on par with traditional clinical rehabilitation treatments [18]. For example, it is shown that with the contribution of online exercises, older adults with spinal cord injury can develop their well-being and experience greater life satisfaction during times like pandemics and when faced with challenges regarding in-person exercise programs [19]. As an example, an online Pilates exercise program was previously shown to improve trunk proprioception and muscle endurance significantly [20].

To the best of our knowledge, no study has been conducted to investigate the effects of telerehabilitation-based corrective exercises on age-related THK, respiratory function, and its other related complications. Therefore, this study aimed to evaluate the effectiveness of 6 weeks of telerehabilitation-based corrective exercises on posture, disability, respiratory function, and quality of life in the elderly with THK.

## Materials & Methods

### Participants

In this randomized clinical study, a total of older adults with THK were randomly assigned to either intervention (N = 20) or control groups (N = 20) based on simple block randomization (by blocking the number of participants on 20 persons in each group). The participants enrolled in the study voluntarily in response to call posters displayed in medical centers for the elderly living in Tabriz city. It was not a prerequisite for the study participants to have been diagnosed with THK before their enrollment. The elderly who were frequent visitors to medical centers for regular health check-ups and showed interest in participating in the study contacted the researchers to inquire about joining the study. Following this, the volunteers were invited to an introductory meeting where they were provided with an overview of the study's inclusion and exclusion criteria and a detailed explanation of the research methods and collaboration procedures. 40 out of 205 volunteers evaluated were selected to participate in the study. This study had no concealment before the allocation of subjects to groups. In this study, the sample size was estimated by employing G*Power version 3.1 based on a previous study investigating the effects of corrective exercises on physical function in both groups [21]. At least a total sample size (n = 22) was required to achieve the 80% statistical power at a 95% confidence interval and by considering the effect size of 1.27. To eliminate the possible effect of dropouts and enhance the statistical power, we decided to recruit 40 participants. A blinded researcher was responsible for randomization by using a table of random numbers. We included adults aged ≥ 60 with a TK angle of more than 40◦ [22] without participation in any regular exercise routines during the past year. The study exclusion criteria were a history of any neuromuscular diseases that affected the participants' balance, osteoporosis diagnosed with the bone densitometry test, presence of back pain, a history of osteoarthritis, falls while walking, cancers and chemotherapy, the long-term use of corticosteroids medication, obvious spinal malalignments according to Nordic screening tool [23], visual or hearing disorders, surgical procedures on spine and lumbopelvic, and reluctance for participating in the experiment. Firstly, the study protocol was approved by the Biomedical Research Ethics Committee of Allameh Tabataba'i University (ATU) (ethical code: IR.ATU.REC.1400.012) and registered with the IRCT registration number: IRCT20180626040244N3, Date: 08/10/2022. All participants gave the written informed consent to participate in the study. The authors confirmed that all research was performed under relevant guidelines/regulations.

In the interview session, the Iranian version of the Short-form 36 Health Status Questionnaire (SF-36) was used to determine the quality of life in the older population. Its reliability was demonstrated by a Cronbach's coefficient in a prior study, ranging from 0.77 to 0.90 [24]. This questionnaire consisted of 36 items evaluating physical performance, physical function limits, general health, happiness, social engagement, psychological health, and well-being. Moreover, the Persian Barthel Index, indicating good validity and reliability, was employed to assess the physical ability of participants. One rater's test–retest resulted in an intraclass correlation coefficient (ICC) of 0.936 (95% CI; 0.895–0.965) [25]. TK angle was measured using a flexible ruler (length: 60cm, width: 2cm) as a noninvasive approach [22], and individuals with TK angle ≥ 40◦ were selected. In this approach, the spinous processes of T2 (as the initial curve point) and T12 vertebras (as the ending curve point) were marked, and the angle between these two landmarks was extracted based on the method used in the previous studies [26]. A previous study demonstrated high-reliability findings for the kyphosis height (0.93, 95% CI: 0.85–0.97) and index of kyphosis (0.89, 95% CI: 0.77–0.94) while using a flexible ruler to measure TKA [27]. Moreover, the photogrammetric method with proven reliability was employed to assess head and neck posture [28]. When utilizing photogrammetric methods to assess head posture, excellent intrarater (95% ICC of 0.98 to 0.99) and interrater (95% ICC, 0.91–0.99) reliabilities were reported [29]. A Nikon camera (D7500) was set on an adjustable camera stand at the height of every person's acromion with an 80 cm distance from them in lateral view [30]. Photos were taken by identifying the C7 vertebra of the neck, the ear tragus of the ear, and the head of the acromion bone in the shoulder using paper glue markers on individuals’ skin. The craniovertebral, cranial, and shoulder angles were obtained using AutoCAD software. We measured the above angles as the craniovertebral angle, formed by intersecting two lines connecting the ear tragus to the C7 vertebra and the horizontal line connecting to the C7 vertebrae. A cranial angle is formed by crossing two lines connecting the ear tragus with the C7 vertebrae and a horizontal line from the ear tragus. A shoulder angle is formed by intersecting two lines connecting the shoulder acromion with the C7 and a horizontal anterior–posterior line [30]. To examine the chest excursion, the diameter of the participant’s chest was measured using a tape meter at the level of the T10 spinous process in maximal exhalation and maximal inhalation phases. The chest expansion was determined by subtracting maximal inhalation from maximal exhalation measures for every participant. Exempt for the questionnaires, each measurement was performed three times, and the mean average of measures was used as the study data. A blinded examiner conducted all measurements in both groups before and after the intervention. The examiner has a Master's degree in Corrective Exercises and has received the necessary training to assess musculoskeletal conditions. The pretest (before starting the study) and posttest (maximally seven days after the last session of the study) measures were examined in an in-person session. Moreover, all data analysis was performed by a statistician blinded from the study group assignments.

### Intervention

The experimental group underwent the corrective exercise protocols with online supervision (Video online WhatsApp chat) for 6 weeks (3 sessions per week × 60 min), augmented with breathing practices. In the first session, all participants in the experimental group were trained in correctly performing the exercises. We also asked the participants to record videos from this session to review the exercises whenever they wanted. In addition, the participants received a research manual with illustrations of optimal neutral spinal alignment when doing functional activities, such as sitting, standing, bending, and sleeping. They were also instructed to practice good posture at least three times daily, aside from study visits. The materials used for this study were given to the participants for free.

**Fig. 1:**
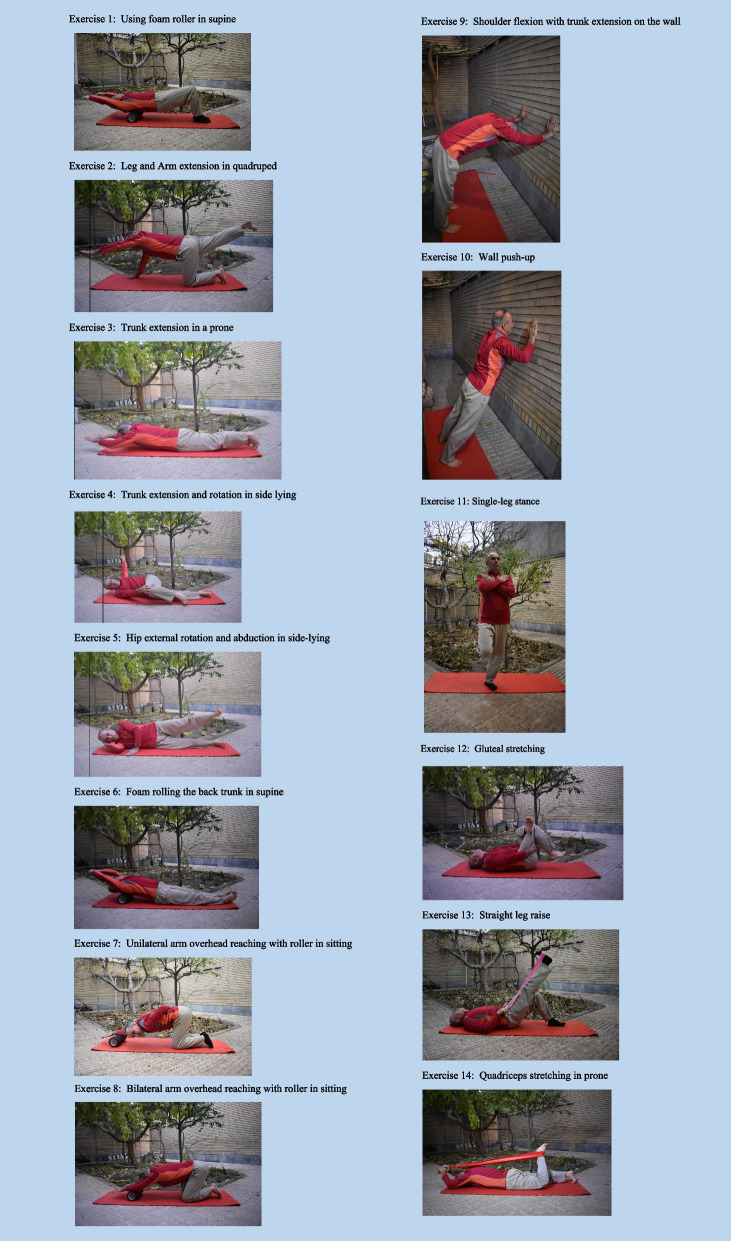
Visual illustrations for prescribed exercises in the elderly with THK

The remaining sessions were performed by online supervision. Each training session included 10 min of general warm-up, 40 min of corrective exercises, and 10 min of cool-down. All exercise sessions were performed under the direct online supervision of the first author. Exercises were used as part of the intervention to address issues with spinal extensor muscle weakness, inadequate recruitment and activation of the muscles, limited spinal mobility, and improper postural alignment. The corrective exercise protocol was according to the previous studies of Katzman et al. 2017 [21, 26], (see Table 1 and Fig. 1). To add breathing exercises to this protocol, the participants were asked to control their breathing so that they inhale while performing exercises, including trunk extension, shoulder girdle adduction, and exhaling in opposite movements. Throughout the trial, exercises progressed with a focus on performing movements in proper pattern and maintaining Borg Scale intensity of 4–5, based on 70–80% of perceived exertion. During the functional exercises, participants were taught to bend at the hips and knees while maintaining neutral spinal alignment with their head over their pelvis and base of support [21]. Moreover, we added specific breathing exercises to the above-mentioned protocol to address respiratory function in the elderly with THK. In the first two weeks of breathing exercises, participants were asked to place their hands on the anterior lunge lobes, extend their inhale and hold it for a while, and then exhale fully. Participants completed 5 repetitions of breathing exercises while performing a corrective exercise routine. After the first two weeks of exercise protocol, patients completed the prescribed exercises without placing their hands on their chest and could complete their routine effortlessly. During the entire exercise sessions, patients were verbally instructed to maintain the ideal alignment, reduce kyphotic posture, and use diaphragmatic breathing.

**Table 1:** A brief description of respiratory and corrective exercises, the aim of exercises, and the duration or frequency of them

No	Type of Exercise	Aim of exercise	Type of Exercises	Duration (s) or Frequency of the exercises	Tools used for exercise progression
1	Using foam roller in supine to activate transversus abdominus	✓ Improve core stability ✓ Improve mobility of the spine and extremities ✓ Strengthen core muscles	Mobility/ Strengthening	10 rep, 1 Set	Roller
2	Leg and Arm extension in a quadruped position	✓ Improve core stability ✓ Improve mobility of the spine and extremities ✓ Strengthen upper back muscles	Strengthening	8 rep, 2 sets	Weight cuffs
3	Trunk extension in a prone position	✓ Improve core stability ✓ Strengthen upper back muscles	Strengthening	8 rep, 2 sets	Weight cuffs
4	Trunk extension and rotation in side lying	✓ Strengthen upper back muscles ✓ Improve upper back mobility	Mobility/ Strengthening	8 rep, 2 sets	Theraband
5	Hip external rotation and abduction in side-lying	✓ Strengthening the hip abductor muscles	Strengthening	8 rep, 2 sets	Weight cuffs
6	Foam rolling the back trunk in supine	✓ Improve spinal extension mobility	Mobility	10 rep, 1 Set	Roller
7	Unilateral arm overhead reaching with roller in kneeling	✓ Improve spinal and chest extension/side flexion mobility	Mobility	10 rep, 1 Set	Roller
8	Bilateral arm overhead reaching with roller in kneeling	✓ Improve spinal and chest extension mobility	Mobility	10 rep, 1 Set	Roller
9	Shoulder flexion with trunk extension on the wall	✓ Improve spinal and shoulder mobility	Mobility	10 rep, 1 Set	Body weight
10	Wall push-up	✓ Scapular stabilization ✓ Shoulder and trunk mobility	Strengthening/Mobility	10 rep, 1 Set	Body weight
11	Single leg stance	✓ Improve postural control	Balance training	10 rep, 1 Set	Body weight
12	Gluteal stretching	✓ Improve mobility of posterior hip capsule and hip extensor muscles	Mobility	Passive 30 s hold, 1 rep	–-
13	Straight leg raise	✓ Stretching the hamstrings and calf muscles	Mobility	Passive 30 s hold, 1 rep	Stretch strap
14	Quadriceps stretching in prone	✓ Stretching the quadriceps muscles	Mobility	Passive 30 s hold, 1 rep	Stretch strap

Breathing pattern: The participants were asked to control their breathing so that they inhale while performing exercises that included trunk extension or shoulder girdle adduction and exhaling in the opposite movements

Postural adjustments: During the entire exercise sessions, patients were instructed to maintain the ideal alignment, reduce kyphotic posture, and use diaphragmatic breathing

The control group was asked to perform 10 min of general warm-up exercises and stretching exercises followed by 40 min of self-selected walking speed. After that, 10 min of general stretching exercises as cool-down were administered. Also, the participants in the control group were in online contact with the first author in all sessions of exercises. After the study was completed, the control group received all exercises provided to the intervention group. The participants in the control group were informed that they had the option to voluntarily participate in the online sessions of the same exercises.

### Statistical analysis

Statistical analysis was performed using the SPSS version 24. Shapiro–Wilk's test was employed to examine the normal distribution of the data. Between-group differences in response to exercises were examined by using the analysis of covariance (ANCOVA). The pre-test measures were set as a covariate to eliminate the possible effects of inter-individual differences. A p-value of *P* ≤ *0.05* was considered statistically significant. Partial Eta Squared was utilized as Effect Size (ES) in this paper.

## Results

The statistical analysis showed that no significant difference was between the two groups in terms of demographic features. The demographic characteristics of participants are shown in Table 2. No dropouts were reported in this study.

**Table 2:** Demographic characteristics of the experimental group (n = 20) and control group (n = 20) (BMI: body mass index, cm: centimeters, kg: kilograms)

Variables	Experimental Group (*N* = 20) Mean ± SD	Control Group (*N* = 20) Mean ± SD
Age (years)	67.50 ± 6.46	69.80 ± 8.19
Height (cm)	160 ± 8.09	164 ± 9.44
Weight (Kg)	82.33 ± 3.98	85.14 ± 5.34
BMI	22.80 ± 4.3	23.07 ± 3.9

Standard deviation, *BMI* Body mass index, *Cm* centimeters, *Kg* Kilograms

Because this research was conducted during the quarantine period caused by the coronavirus, the subjects had enough time to participate in the classes. Each subject had to participate in classes 3 sessions a week and their schedule was flexible in terms of the hours of the program during the day, so in coordination with the examiner, the subjects held all the training sessions. The ANCOVA results showed that statistically significant differences were found between experimental and control groups concerning time*group on quality of life (*P* < *0.001*) and chest expansion (*P* < *0.001*). However, no statistically significant difference was observed between both groups in terms of physical ability (*P* = *0.251*). The results are summarized in Table 3.

**Table 3:** ANCOVA results comparing the mean average of posttest measures by eliminating possible effects of pretest measures of quality of life, chest expansion, and physical ability for the experimental group (n = 20) and control group (n = 20)

Variable	The experimental **g**roup **(Mean ± SD)**	Control group **(Mean ± SD)**	**F**	*P-value*	ES
QOL (pretest)	66.14 ± 11.64	61.61 ± 9.51	29.08	< 0.001^*^	0.44
QOL (posttest)	67.12 ± 11.61	61.40 ± 8.97			
Chest expansion (pretest)	2.53 ± 0.86	2.94 ± 0.80	47.61	< 0.001^*^	0.56
Chest expansion (posttest)	3.43 ± 0.70	2.74 ± 0.67			
Physical ability (pretest)	71.19 ± 11.21	72.68 ± 9.50	1.36	0.251	0.04
Physical ability (posttest)	70.62 ± 10.46	72.71 ± 9.43			

*QOL* Quality of life, *SD* Standard Deviation

^*^Statistically significant differences observed, ES: Effect size

Regarding postural variables, the results demonstrated that THK (*P* < *0.001*), craniovertebral (*P* < *0.001*), cranial (*P* < *0.001*), and shoulder (*P* = *0.005*) angles were significantly improved in the experimental group as compared with controls. Table 4 presents more information.

**Table 4:** ANCOVA results comparing the mean average of posttest measures by eliminating possible effects of pretest measures for postural measures for the experimental group (n = 20) and control group (n = 20)

Variable	The experimental **g**roup **(Mean ± SD)**	Control group **(Mean ± SD)**	**F**	*P*-value	ES
CVA (pretest)	44.13 ± 5.03	45.90 ± 4.79	18.57	< 0.001^*^	0.33
CVA (posttest)	45.64 ± 4.78	45.79 ± 4.62			
SA (pretest)	41.63 ± 3.73	40.88 ± 3.94	8.94	0.005^*^	0.20
SA (posttest)	39.98 ± 3.74	40.59 ± 3.40			
**TK**(pretest)	48.55 ± 2.58	48.98 ± 2.11	16.50	< 0.001^*^	0.31
TK (posttest)	47.08 ± 2.24	48.92 ± 2.26			
CA (pretest)	21.26 ± 3.92	20.46 ± 3.49	22.77	< 0.001^*^	0.38
CA (posttest)	22.51 ± 3.71	20.69 ± 2.97			

All measurement scales are in degrees

*CVA* Craniovertebral Angle, *SA* Shoulder Angle, *TK* Thoracic kyphosis, *CA* Cranial angle, *SD* Standard Deviation

^*^Statistically significant differences observed, ES: Effect size

## Discussion

The results of the current study demonstrated that six weeks of telerehabilitation-based respiratory and corrective exercises improved trunk and shoulder posture, and quality of life in the experimental group, in addition to helping the elderly with limited lung capacity due to THK to increase their chest expansion compared to a control group. However, no significant difference was observed between both groups in terms of the physical ability questionnaire.

Regarding postural alignment, our results showed that the craniovertebral, cranial, and shoulder angles significantly improved after six weeks of online respiratory and corrective exercises. Our findings are in line with those of previous studies suggesting that telerehabilitation exercises significantly improved postural deviation [31–33]. Thus far, many studies have evaluated the significant effects of different exercise programs, such as therapeutic exercises [34], scapular stabilization [35], and strengthening exercises [36] on the neck, shoulder, and head posture. However, our results are not consistent with some of the previous studies showing the ineffectiveness of exercise therapy on a cranial angle. Previous studies found that forward head posture is significantly modified by prescribing corrective exercises [34, 37]. Moreover, a recent study showed that feedback-based online training can enhance postural metrics in adolescent soccer players for six weeks [38]. This improvement may be due to the effect of exercises focused on the back on minimizing the upper trapezius and maximizing the lower trapezius activation [37, 39]. It seems that age-related hyperkyphosis can be managed through technology-based exercise and posture training, which may be more widely disseminated than in-person training [40].

Regarding respiratory patterns, the results of the current study indicated that corrective exercises could improve chest expansion in older adults. Recently, the use of telerehabilitation has been recommended for the management of different pulmonary dysfunctions [41, 42]. Our results are consistent with those of a previous study suggesting that thorax-focused corrective exercises can improve breathing and chest expansion in older adults with THK [13]. Moreover, the use of corrective exercises for improving respiratory capacity in patients with spinal cord malalignments has been investigated in several studies [13, 43]. For instance, a study indicated that six weeks of an exercise protocol, including stretching, strengthening, and stability exercises with a Physioball, could improve chest expansion in female computer users with Upper Crossed Syndrome [43]. Consistent with this finding, it is shown that telerehabilitation trials may improve exercise capacity and respiratory function without any known negative effects when compared to the no rehabilitation control [44]. This improvement may be due to adopting a more efficient trunk muscle activation strategy, increased breathing rhythm control, and decreased muscle tension resulting from respiratory and corrective exercises [13, 45].

In line with previous studies [46, 47], our findings showed that prescribing respiratory and corrective exercises improved QOL in the study participants. It seems that the rehabilitation exercise program generally had favorable effects on QOL as the participants' perceptions of their QOL were improved by encouraging their physical and mental health, which is consistent with previous studies [31, 33, 42, 48, 49].

Of note is that in the current study, the combination of respiratory and corrective exercise protocols significantly improved the quality of life in older adults with THK. Several studies have reported the same beneficial results after corrective protocols on the well-being of older adults [50, 51]. Recent evidence indicated that as compared with other interventions, it appears that exercise through telerehabilitation may be an alternative to enhance the quality of life in persons with physical disabilities and medical illnesses [50, 52]. The underlying mechanism behind this improvement is the positive effects of exercise programs on spinal balance, back muscle strength, and walking performance [51].

With a high prevalence of THK progression in older adults because of age-related diseases, including osteoporosis, vertebral fractures [26], bone loss, degenerative disk diseases, loss of muscle strength, and limited mobility [53], designing efficient exercise programs to minimize THK complications becomes of paramount importance. However, the eagerness to participate in conventional exercise programs may decrease this population due to several factors, particularly pain, discomfort, or the COVID-19 pandemic [14]. Thus, technology can help them adhere to their prescribed exercise routine. For example, implementing an online exercise program via Zoom application improved physical activity in the elderly without the risk of confronting during quarantine while increasing their interest in adhering to this protocol notably [54]. Thus it seems that online exercises can be helpful in conditions where there is no chance for conventional programs.

The findings, from using the Partial eta squared method indicate that the exercise program impact size in the ANCOVA test falls within the range of 0.2 to 0.4. This suggests an effect size signifying that the online corrective exercises influenced the parameters we measured. It is important to delve into what this range of effect sizes means for our study. The moderate effect size implies that the online corrective exercises had a clinically relevant impact on aspects of elderly individuals with thoracic hyperkyphosis, such as their QOL, chest expansion, and postural angles. These results lend support to the practicality and effectiveness of implementing targeted interventions, for this population. However further investigations are necessary to comprehend the long-term consequences and broader health benefits associated with these exercises.

However, this study has some limitations. Firstly, the current study was conducted on older adults, and thus the results cannot be generalized to all aging populations, including the younger ones. Secondly, the study participants were healthy adults without any pain or disease, including osteoporosis, stroke, and balance impairment, so the outcomes may differ in these populations. Thirdly, one of the other limitations of this study is measuring variables immediately after six weeks of the exercise program and not completing follow-up assessments to evaluate the durability of improvements. Lastly, it has been planned to examine respiratory function with the spirometry method. However, because of the COVID-19 pandemic and the high risk of infection, we decided to replace chest expansion with it.

## Conclusion

Prescribing telerehabilitation corrective exercises can improve spinal alignment, quality of life, and chest expansion in the elderly with THK. Due to the limitations of participating in face-to-face exercise sessions, particularly during crises like the COVID-19 pandemic, online exercises may be replaced by the elderly population. It is therefore recommended to combine exercises aimed at improving and modifying respiratory function with corrective exercises for tackling the effects of THK on neck-shoulder posture, lung capacity, balance, and quality of life.

### Supplementary Information

**Additional file 1: Supplementary1. **The raw data and material analyzed in this study.

## Data Availability

All data generated or analyzed during this study are included in this study and its supplementary information files (Supplementary File 1).

## References

[1] Lutz W, K C S. Dimensions of global population projections: what do we know about future population trends and structures? Philos Trans R Soc Lond B Biol Sci. 2010;365(1554):2779–91. 10.1098/rstb.2010.0133. 10.1098/rstb.2010.0133 PMC2935115 20713384

[2] Harper S (2014). Economic and social implications of aging societies. Science.

[3] Lewis R, Gómez Álvarez CB, Rayman M, Lanham-New S, Woolf A, Mobasheri A (2019). Strategies for optimising musculoskeletal health in the 21(st) century. BMC Musculoskelet Disord.

[4] Bassani T, Galbusera F, Luca A, Lovi A, Gallazzi E, Brayda-Bruno M (2019). Physiological variations in the sagittal spine alignment in an asymptomatic elderly population. Spine J.

[5] Koelé MC, Lems WF, Willems HC (2020). The Clinical Relevance of Hyperkyphosis: A Narrative Review. Front Endocrinol.

[6] Katzman WB, Wanek L, Shepherd JA, Sellmeyer DE (2010). Age-related hyperkyphosis: its causes, consequences, and management. J Orthop Sports Phys Ther.

[7] Benedetti MG, Berti L, Presti C, Frizziero A, Giannini S (2008). Effects of an adapted physical activity program in a group of elderly subjects with flexed posture: clinical and instrumental assessment. J Neuroeng Rehabil.

[8] Huang M-H, Barrett-Connor E, Greendale GA, Kado DM (2006). Hyperkyphotic Posture and Risk of Future Osteoporotic Fractures: The Rancho Bernardo Study. J Bone Mineral Res.

[9] Naderi A, Rezvani MH, Shaabani F, Bagheri S (2019). Effect of Kyphosis Exercises on Physical Function, Postural Control and Quality of Life in Elderly Men With Hyperkyphosis. Salmand Iran J Ageing.

[10] Huang YH, Fang IY, Kuo YL (2021). The Influence of Nordic Walking on Spinal Posture, Physical Function, and Back Pain in Community-Dwelling Older Adults: A Pilot Study. Healthcare.

[11] González-Gálvez N, Gea-García GM, Marcos-Pardo PJ (2019). Effects of exercise programs on kyphosis and lordosis angle: a systematic review and meta-analysis. PLoS ONE.

[12] Bansal S, Katzman WB, Giangregorio LM (2014). Exercise for improving age-related hyperkyphotic posture: a systematic review. Arch Phys Med Rehabil.

[13] Jang HJ, Kim MJ, Kim SY (2015). Effect of thorax correction exercises on flexed posture and chest function in older women with age-related hyperkyphosis. J Phys Ther Sci.

[14] Aksay E (2021). Live online exercise programs during the Covid-19 pandemic–are they useful for elderly adults?. Journal of Physical Education and Sport.

[15] Rabanifar N, Abdi K (2021). Barriers and Challenges of Implementing Telerehabilitation: A Systematic Review. Iran Rehabil J.

[16] Kreider CM, Hale-Gallardo J, Kramer JC, Mburu S, Slamka MR, Findley KE (2022). Providers' shift to telerehabilitation at the U.S. veterans health administration during Covid-19: practical applications. Front Public Health.

[17] Zheng J, Hou M, Liu L, Wang X (2022). Knowledge Structure and Emerging Trends of Telerehabilitation in Recent 20 Years: A Bibliometric Analysis via CiteSpace. Front Public Health.

[18] Anil K, Freeman JA, Buckingham S, Demain S, Gunn H, Jones RB (2021). Scope, context and quality of telerehabilitation guidelines for physical disabilities: a scoping review. BMJ Open.

[19] Mehta S, Ahrens J, Abu-Jurji Z, Marrocco SL, Upper R, Loh E (2021). Feasibility of a virtual service delivery model to support physical activity engagement during the COVID-19 pandemic for those with spinal cord injury. J Spinal Cord Med.

[20] Suner-Keklik S, Numanoglu-Akbas A (2022). An online pilates exercise program is effective on proprioception and core muscle endurance in a randomized controlled trial. Ir J Med Sci.

[21] Katzman WB, Vittinghoff E, Lin F, Schafer A, Long RK, Wong S (2017). Targeted spine strengthening exercise and posture training program to reduce hyperkyphosis in older adults: results from the study of hyperkyphosis, exercise, and function (SHEAF) randomized controlled trial. Osteoporos Int.

[22] Greendale G, Nili N, Huang M-H, Seeger L, Karlamangla A (2011). The reliability and validity of three non-radiological measures of thoracic kyphosis and their relations to the standing radiological Cobb angle. Osteoporos Int.

[23] Crawford JO (2007). The Nordic musculoskeletal questionnaire. Occup Med.

[24] Montazeri A, Goshtasebi A, Vahdaninia M, Gandek B (2005). The Short Form Health Survey (SF-36): Translation and validation study of the Iranian version. Qual Life Res.

[25] Hormozi S, Alizadeh-Khoei M, Sharifi F, Taati F, Aminalroaya R, Fadaee S (2019). Iranian Version of Barthel Index: Validity and Reliability in Outpatients' Elderly. Int J Prev Med.

[26] Roghani T, Zavieh MK, Manshadi FD, King N, Katzman W (2017). Age-related hyperkyphosis: update of its potential causes and clinical impacts-narrative review. Aging Clin Exp Res.

[27] Yanagawa TL, Maitland ME, Burgess K, Young L, Hanley D (2000). Assessment of Thoracic Kyphosis Using the Flexicurve for Individuals with Osteoporosis. Hong Kong Physiother J.

[28] Singla D, Veqar Z, Hussain ME (2017). Photogrammetric Assessment of Upper Body Posture Using Postural Angles: A Literature Review. J Chiropr Med.

[29] Gadotti IC, Armijo-Olivo S, Silveira A, Magee D (2013). Reliability of the Craniocervical Posture Assessment: Visual and Angular Measurements Using Photographs and Radiographs. J Manipulative Physiol Ther.

[30] Heydari Z, Sheikhhoseini R (2022). Establishing minimal clinically important difference for effectiveness of corrective exercises on craniovertebral and shoulder angles among students with forward head posture: a clinical trial study. BMC Pediatr.

[31] Lee Y, Jung KB (2022). Effect of Physiotherapy to Correct Rounded Shoulder Posture in 30 Patients During the COVID-19 Pandemic in South Korea Using a Telerehabilitation Exercise Program to Improve Posture, Physical Function, and Reduced Pain, with Evaluation of Patient Satisfaction. Medi Sci Monit.

[32] Muñoz-Tomás MT, Burillo-Lafuente M (2023). Telerehabilitation as a Therapeutic Exercise Tool versus Face-to-Face Physiotherapy: A Systematic Review. Int J Environ Res Public Health.

[33] Moulaei K, Sheikhtaheri A, Nezhad MS, Haghdoost A, Gheysari M, Bahaadinbeigy K (2022). Telerehabilitation for upper limb disabilities: a scoping review on functions, outcomes, and evaluation methods. Archives of Public Health.

[34] Sheikhhoseini R, Shahrbanian S, Sayyadi P, O'Sullivan K (2018). Effectiveness of Therapeutic Exercise on Forward Head Posture: A Systematic Review and Meta-analysis. J Manipulative Physiol Ther.

[35] Im B, Kim Y, Chung Y, Hwang S (2016). Effects of scapular stabilization exercise on neck posture and muscle activation in individuals with neck pain and forward head posture. J Phys Ther Sci.

[36] Kim JY, Kwag KI (2016). Clinical effects of deep cervical flexor muscle activation in patients with chronic neck pain. J Phys Ther Sci.

[37] Go SU, Lee BH (2016). Effects of scapular stability exercise on shoulder stability and rehabilitative ultrasound images in office workers. J Phys Ther Sci.

[38] Ludwig O, Dindorf C (2022). Effects of Feedback-Supported Online Training during the Coronavirus Lockdown on Posture in Children and Adolescents. J Funct Morphol Kinesiol.

[39] Day JM, Uhl T (2013). Thickness of the lower trapezius and serratus anterior using ultrasound imaging during a repeated arm lifting task. Man Ther.

[40] Katzman WB, Gladin A, Lane NE, Wong S, Liu F, Jin C (2019). Feasibility and Acceptability of Technology-Based Exercise and Posture Training in Older Adults With Age-Related Hyperkyphosis: Pre-Post Study. JMIR Aging.

[41] Lippi L, D'Abrosca F (2022). Closing the Gap between Inpatient and Outpatient Settings: Integrating Pulmonary Rehabilitation and Technological Advances in the Comprehensive Management of Frail Patients. Int J Environ Res Public Health.

[42] Malik S, Dua R, Krishnan AS, Kumar S, Kumar S, Neyaz O (2022). Exercise Capacity in Patients With Chronic Obstructive Pulmonary Disease Treated With Tele-Yoga Versus Tele-Pulmonary Rehabilitation: A Pilot Validation Study. Cureus.

[43] Javazi F, Sedaghati P, Daneshmandi H (2019). The Effect of Selected Corrective Exercises With Physioball on the Posture of Female Computer Users With Upper Crossed Syndrome. Journal of Sport Biomechanics.

[44] Cox NS, Dal Corso S, Hansen H, McDonald CF, Hill CJ, Zanaboni P (2021). Telerehabilitation for chronic respiratory disease. Cochrane Database Syst Rev.

[45] Kava KS, Larson CA, Stiller CH, Maher SF (2010). Trunk endurance exercise and the effect on instrumental performance: a preliminary study comparing Pilates exercise and a trunk and proximal upper extremity endurance exercise program. Music Performance Research.

[46] Moreira S, Criado MB, Ferreira MS, Machado J (2022). Positive Effects of an Online Workplace Exercise Intervention during the COVID-19 Pandemic on Quality of Life Perception in Computer Workers: A Quasi-Experimental Study Design. Int J Environ Res Public Health.

[47] Kim S, Yi D, Yim J (2022). The effect of core exercise using online videoconferencing platform and offline-based intervention in postpartum woman with diastasis recti abdominis. Int J Environ Res Public Health.

[48] Ajmera P, Miraj M, Kalra S, Goyal RK, Chorsiya V, Shaik RA (2022). Impact of telehealth interventions on physiological and psychological outcomes in breast cancer survivors: A meta-analysis of randomised controlled trials. Front Oncol.

[49] Granet J, Peyrusqué E, Ruiz F, Buckinx F, Abdelkader LB, Dang-Vu TT (2023). Online physical exercise intervention in older adults during lockdown: Can we improve the recipe?. Aging Clin Exp Res.

[50] Jang HJ, Hughes LC, Oh DW, Kim SY. Effects of Corrective Exercise for Thoracic Hyperkyphosis on Posture, Balance, and Well-Being in Older Women: A Double-Blind, Group-Matched Design. J Geriatr Phys Ther. 2019;42(3):E17–E27. 10.1519/JPT.0000000000000146. 10.1519/JPT.0000000000000146 28914720

[51] Imagama S, Hasegawa Y, Matsuyama Y, Sakai Y, Ito Z, Hamajima N (2011). Influence of sagittal balance and physical ability associated with exercise on quality of life in middle-aged and elderly people. Arch Osteoporos.

[52] Dias JF, Oliveira VC, Borges PRT, Dutra F, Mancini MC, Kirkwood RN (2021). Effectiveness of exercises by telerehabilitation on pain, physical function and quality of life in people with physical disabilities: a systematic review of randomised controlled trials with GRADE recommendations. Br J Sports Med.

[53] Katzman W, Vittinghoff E, Kado D (2011). Age-related hyperkyphosis, independent of spinal osteoporosis, is associated with impaired mobility in older community-dwelling women. Osteoporos Int.

[54] Schwartz H, Har-Nir I, Wenhoda T, Halperin I (2021). Staying physically active during the COVID-19 quarantine: exploring the feasibility of live, online, group training sessions among older adults. Transl Behav Med.

